# Community-engagement in research in humanitarian settings

**DOI:** 10.3389/fpubh.2023.1208684

**Published:** 2023-08-17

**Authors:** Luchuo Engelbert Bain, Claude Ngwayu Nkfusai, Prudence Nehwu Kiseh, Oluwaseun Abdulganiyu Badru, Lundi Anne Omam, Oluwafemi Atanda Adeagbo, Ikenna Desmond Ebuenyi, Gift Malunga, Eugene Kongnyuy

**Affiliations:** ^1^Department of Psychology, Faculty of Humanities, University of Johannesburg, Johannesburg, South Africa; ^2^Triangle Research Foundation (TRIFT), Limbe, Cameroon; ^3^Global South Health Services and Research (GSHS), Paris, France; ^4^Department of Public Health, School of Nursing and Public Health, University of KwaZulu-Natal, Durban, South Africa; ^5^Clinton Health Access Initiative, Yaoundé, Cameroon; ^6^Cameroon Baptist Convention Health Services, Bamenda, Cameroon; ^7^Usmanu Danfodiyo University Teaching Hospital, Sokoto, Nigeria; ^8^Institute of Human Virology, Abuja, Nigeria; ^9^Department of Public Health and Primary Care, University of Cambridge, Cambridge, United Kingdom; ^10^Department of Community and Behavioral Health, College of Public Health, University of Iowa, Iowa City, IA, United States; ^11^Department of Sociology, Faculty of Humanities, University of Johannesburg, Auckland Park, South Africa; ^12^Department of Rehabilitation Science and Technology, University of Pittsburgh, Pittsburgh, PA, United States; ^13^United Nations Population Fund, Lusaka, Zambia; ^14^United Nations Population Fund, Kinshasa, Democratic Republic of Congo

**Keywords:** community engagement, research, humanitarian settings, ethical consideration, gender issues

## Background

Armed conflict, natural disasters, and forced relocation are among the humanitarian problems affecting hundreds of millions of people worldwide (1, 2). Almost 80 million people have been evicted from their homes against their will (3), and one in every six children lives in or near a combat zone (4). Humanitarian emergencies may arise due to natural disasters (e.g., earthquake in Syria and Turkey) and manmade/technological disasters (e.g., Ukraine, Russia, Yemen, Syria, and South Sudan) (4). Also, humanitarian emergencies may be indicated in cases of forced displacements, as evident with the inflow of refugees into Lebanon and Bangladesh, and could be internal displacement, as seen in the Democratic Republic of Congo, Ethiopia and most recently, Cameroon (2). Additionally, complex emergencies (co-occurrence of natural and manmade) may necessitate urgent humanitarian emergencies to curb immediate and future public health issues (2). Complex emergencies can occur when there is a breakdown in authority, an attack on a critical location, looting, numerous other conflict situations, or even wars (2). Some countries are coping with many crises simultaneously or protracted crises that have lasted for decades (5).

Few studies have examined the difficulties in forming solid and balanced research partnerships, despite the limited or lack of research in such settings being widely discussed over the past decade (6). Methodological constraints, ethical concerns, security issues, and logistical challenges have been identified as the main barriers to conducting research in such settings (7). Populations affected by conflict and other crises confront a variety of humanitarian issues and demands, such as population relocation, family separation, food shortages, a lack of health care, and increased susceptibility to outbreaks (8, 9). The term “health research in humanitarian crises” refers to medical research during humanitarian crises and/or research on a crisis-inflicted population (such as a population of refugees who have fled a violent conflict and have been relocated to a more peaceful area) (10). Collaboration with humanitarian organizations is crucial if research findings are to be implemented and incorporated into humanitarian policies and procedures; this is particularly important to address logistic and security difficulties (11).

## Why is research in humanitarian research needed?

Humanitarian needs are numerous and varied (1). Research in humanitarian situations is gaining traction and is now a worthwhile endeavor that permits the generation of contextually suitable knowledge to respond to the needs of those impacted by humanitarian disasters (1). Health research is necessary to guarantee that humanitarian response to disasters is evidence-based, effective, and establishes the foundation for post-crisis health planning, implementation, and evaluation (1, 4). It is believed that it is unethical to refrain from undertaking research in war and crisis circumstances, but contextual-based research is needed to help manage these crises (4). For instance, developing information and evidence about how to best care for children in emergency situations is vital (4). Children are being displaced and caught up in wars in greater numbers every year. They are the most vulnerable to climate change and environmental deterioration and frequently face famine risks (12). Children are increasingly confronted with long-lasting crises and unstable environments. Since 2010, United Nations Children's Fund (UNICEF) has responded to 300 humanitarian emergencies annually in around 90 countries (12).

Research can be used to understand and address problems in humanitarian settings (12, 13). Given the busy environment in which many humanitarian workers work, practitioners will be aware of these to some extent based on their experience, but given that, there is frequently little time to fully absorb the most recent conversations among practitioners, academics, policymakers, and civil society (13). This knowledge gap can be filled through research in the field, which will also assist practitioners in staying up to date with ongoing discussions about development and humanitarian aid (12). Researchers play a crucial role in translating complex information, such as observations, derived learnings from settings, research evidence, and specialized terminology, into a format that can be easily understood by practitioners and decision-makers who may not have an academic background or familiarity with academic traditions (11–13). Neglecting to utilize the knowledge gained from humanitarian contexts to enhance practice and services would be a missed opportunity (13, 14). However, it is important to emphasize that research should uphold ethical principles and avoid exploiting the vulnerability of individuals in these settings, aligning with the guidelines laid out in the Helsinki Declaration (13–15).

## Why should vulnerable populations be engaged in research?

Humanitarian crises are made more vulnerable by inequalities and hierarchies in age, gender, social class, religion, and race because they restrict individuals from achieving their fundamental needs, accessing resources, and asserting their rights (14). Power asymmetry between research and study participants is heightened in these settings because of the several needs of individuals in these settings (15). Since the vulnerability needs to be investigated, especially in humanitarian settings, many researchers find themselves using a vulnerable population (15). For instance, research may be required to comprehend the vulnerability, address the therapy of, enhance the environment for, or alter policies in support of a specific ailment (14).

When it comes to receiving humanitarian relief, vulnerability—situation of being susceptible to physical or emotional harm—becomes a tool for human agency and a negotiating chip (16). Vulnerability can therefore influence social behaviors in a beneficial way. During humanitarian crises, one should act in ways that promote more openness and allow disadvantaged groups access to public spaces, especially when the impacted people is working together to rebuild their society (16). The condition of human interdependence, relational life, ties to others, and shared humanity is described here as vulnerability (17). Understanding the people, especially vulnerable populations, is crucial for reacting to humanitarian crises (12). Therefore, vulnerable groups should be involved in robust research that can ultimately increase their living condition and quality of life.

## Community engagement, barriers and facilitators to effective research in humanitarian settings

Community engagement is the act of interacting with and through groups of people who are connected by proximity, shared interests, or similar circumstances in order to address issues impacting the welfare of the populace. Community engagement is an effective tool for behavioral and environmental change to improve residents' and neighborhood's health (17–19). Partnerships and coalitions with relevant key humanitarian players are frequently employed in this process because to their capacity to mobilize resources, influence systems, alter partner relationships, and function as catalysts for changing programs, policies, and practices (18, 19). Bain et al. (20), proposed a framework for effective community engagement in research. They suggested some key elements of community-engaged research such as mutual respect, community motivation, community empowerment, and co-creation of interventions with communities, adherence to appropriate ethical principles, and gender mainstreaming. Table 1 shows barriers, facilitators and the reasons for community-engaged research in humanitarian settings as well as key recommendations.

**Table 1:** Community-engagement in research in humanitarian settings: barriers, facilitators and recommendations (Source: Authors experiences).

**Why is engaging communities in research in conflict-affected an important subject?**	**Barriers**	**Facilitators**	**Key recommendations for effective community engagement**
Engaging communities facilitates local ownership of humanitarian response, including sustainable homegrown preventive solutions (4)	Increased poverty and food insecurity, including loss of livelihoods during humanitarian situations, leads to low research output and quality as participants cannot give full details needed during research Increased vulnerability due to humanitarian crises	Using locally grown solutions is cheaper and more sustainable in humanitarian preparedness and response Access to resources and commodities	Involvement and participation of community leaders in research so as to have quality outputs and outcomes as they master the terrain more Strengthening local capacities and systems for humanitarian preparedness, resilience and enhancing sustainability
Strengthening humanitarian research with communities, particularly women-led organizations, enhance their capacity to participate in humanitarian research	Limited knowledge and capacity of communities and local organizations to prevent and/or respond to humanitarian issues	Local organizations are the first respondents in humanitarian situations	Engaging and building the capacity of local organizations to design and implement gender-responsive humanitarian programmes, including data collection to guide humanitarian responses
Identification of vulnerable population groups	During humanitarian situations, vulnerable populations such as women and girls suffer more	When equipped with knowledge, communities can take deliberate steps to protect vulnerable populations Use of community-led protection systems for vulnerable population groups	Educating/sensitizing communities on the importance of protecting vulnerable groups, especially women, children, and the older adults Identifying and strengthening potential protection systems can help to reduce women's and children's vulnerability
	Increased poverty and food insecurity, including loss of livelihoods during humanitarian situations Limited mobility during humanitarian situations Increased vulnerability due to humanitarian crises	The use of locally grown solutions is cheaper and more sustainable in humanitarian preparedness and response Access to resources and commodities	Involvement and participation of community leaders in humanitarian situations Strengthening local capacities and systems for humanitarian preparedness, resilience and enhancing sustainability
Strengthening humanitarian work with communities, particularly women-led organizations, enhances their capacity to respond to humanitarian issues	Limited knowledge and capacity of communities and local organizations to prevent and/or respond to humanitarian issues	Local organizations are the first respondents in humanitarian situations	Engaging and building the capacity of local organizations to design and implement gender-responsive humanitarian programmes, including data collection to guide humanitarian responses
Identification of vulnerable population groups	During humanitarian situations, vulnerable population groups such as women and girls suffer more	When equipped with knowledge, communities can take deliberate steps to protect vulnerable populations Use of community-led protection systems for vulnerable population groups	Educating/sensitizing communities on the importance of protecting vulnerable groups, especially women, children and the older adults Identifying and strengthening potential protection systems that can help to reduce women's and children's vulnerability

## Ethical, community engagement, and gender dimensions in humanitarian research

### Ethical considerations during research in humanitarian settings

Humanitarian action is defined as any impartially carried out assistance, protection, or advocacy effort in response to a human need brought on by a complex political emergency or a natural disaster (ALNAP) (21). Delivering services to communities that are in immediate danger is what is referred to as humanitarian aid (21). There is increased interest in conducting research in humanitarian contexts, with studies ranging from population surveys to qualitative evaluations to analyse the complete range of humanitarian relief provisions (1). Medical and health policy research is scarce, and it is frequently undertaken by available international non-governmental organizations (NGOs) and humanitarian relief groups (22). Due to the possibility that this population may be particularly vulnerable to coercion and inadequate consent processes, the public health community has been working hard to uphold ethical principles like justice, autonomy, beneficence, and non-maleficence (21). Traditional ethics review is unworkable because humanitarian urgencies demand prompt assessment and management (1, 21, 22). A key element of research ethics is independent ethics evaluation before the start of a project (22). While the research could be put up alongside aid and plans to address the area's health objectives, conducting research in humanitarian situations is challenging from an ethical standpoint due to the work's time-sensitive nature and complex cultural and security factors (1). The ethical challenges arise when people are exploited or when there is no oversight in terms of the needs of the individual or actors take advantage of affected individuals, e.g., researchers may fail to get ethics approval or have unqualified or untrained people to collect data; lack of appropriate referral or debrief for people that experience psychological distress (1).

Because resource-poor communities in conflict frequently experience unstable political environments and lack adequate technical and scientific guidance to oversee research ethics, international organizations frequently employ different ethical standards that may not comply with human rights or humanitarian laws (1, 22, 23). The sort of ethical assessment will vary depending on the methodology, the specific populations involved, and whether the project entails hypothesis testing, clinical research, or routine monitoring and evaluation (22). In humanitarian healthcare, emphasizing ethical practices might be problematic if it does not consider the perception of the national healthcare staff (1). Healthcare workers believe saving as many lives as possible is good and that intervening with binding ethical codes like seeking consent may waste precious time. Taking away a victim's autonomy can be justified on the grounds that their momentary shock from the accident has impaired their ability to make decisions (23).

It is important to remember that those who occupy positions of authority and responsibility for the benefit of the general welfare also bear positive obligations, such as providing decent housing, reducing inequality, and putting preparatory measures in place to minimize suffering before and after disasters (24). According to a recently published paper by Luchuo et al., the research team, community members, and potential research volunteers must discuss any apparent ethical problems that may arise throughout the study. In-depth community engagement is increasingly seen as the key factor influencing the adoption of effective research, innovations, and interventions. Communities need to be involved in research, according to community leaders, policymakers, and funders (25).

### Community factors (community engagement and community health workers) in research in humanitarian settings

Crisis-affected populations are especially population at risk (2). Every health research should involve affected populations and local communities because it fosters community trust, enhances research designs, and creates a channel for information distribution, leading to higher-quality studies and more useful conclusions (10). Local partners are more connected to local communities because they understand the cultural and historical context, and their involvement enhances the quality and relevance of research (2, 3). Community involvement is crucial for the success of the research's implementation and adoption of its findings after it is finished, in addition to being required ethically (26). It becomes even more important when assisting those experiencing severe trauma and agitation brought on by a humanitarian disaster (27). A key component of a comprehensive strategy for community involvement is community engagement, an increasingly important requirement for research programs (9). Unfortunately, there is little community involvement and little involvement of scholars from crisis-affected nations and regions (9). Community health workers (CHWs) can contribute critically to emergency response and preserve important services during crises by participating in research, particularly in humanitarian circumstances.

With the current COVID-19 pandemic and the West Africa Ebola outbreak, there is a rising understanding of the need of CHWs for the security and resilience of the global health system (11). There is strong evidence that CHWs can provide a variety of services locally, expand access to necessary healthcare services, and that supporting community-based initiatives can result in appreciable gains in neonatal, child, and maternal health (10). Humanitarian organizations addressing the current issue frequently collaborate with researchers conducting humanitarian studies (27). Although significant service delivery limitations may exist during acute crises and protracted times of violence and instability, CHWs can still provide services. They also frequently educate humanitarians about the safety of their region, easing aid delivery processes (11).

The importance of considering community beliefs and culture when conducting research and involving the community in the design and implementation of the study has been stressed by the Médecins Sans Frontières (MSF) ethical committee (11). Health workers will be forced to evacuate during conflicts since many health facilities will be destroyed, looted, or burned down. In response, CHWs, who are typically dependable members of the community, will be sought (6, 28, 29). Despite their critical role, there may be few or no CHWs after the acute crisis phase, which fosters distrust (1).

Determining what and who the community is, as well as who has the authority to speak on its behalf, is a problem that arises in all initiatives aiming at including the community (11). Through community-based surveillance (CBS), the community can also be involved. A community member's systematic observation and reporting of incidents with public health implications is known as CBS (27). These communities are frequently employed in conflict-affected areas and communities that are topographically difficult to reach. In a study conducted in Cameroon utilizing CBS, Metuge et al. (5) discovered that it could aid in the identification of outbreaks as well as other events during crises. Improving the capability of academic and non-governmental organizational-based researchers in and from humanitarian crisis-affected countries could enhance readiness and resilience for future disasters. Community engagement is crucial (27). According to Shanks et al. (10), extensive community participation and community consultation before doing research in humanitarian situations can lessen the threats to these vulnerable groups' autonomy posed by researchers from humanitarian agencies. Although the literature on global health strongly supports community involvement in any research, there are few recommendations for overcoming obstacles in humanitarian circumstances (18).

### Gender considerations in research in humanitarian settings

In humanitarian circumstances, the risk that a woman would experience intimate partner or non-partner violence grows to around 1 in 3 over the course of her lifetime as the perpetrator attacker is brought closer to the victim (7). The peculiar nature of humanitarian settings increases likelihood of sexual based violence (27). Gender-based violence (GBV) is when a person is armed based on their gender expression or identity. Examples of GBV include intimate partner violence (IPV), sexual assault, sexual abuse, economic exploitation, reproductive coercion, and child marriages (8). The negative effects of gender-insensitive pandemic control measures like lockdowns during the most recent coronavirus disease (COVID-19) increased the risk of GBV by bringing victims and survivors closer to abusers, reinforcing gender roles in the home, amplifying socioeconomic stress, restricting access to reproductive and sexual healthcare, all of which were exacerbated in humanitarian circumstances (9). Access to psychosocial support, healthcare and abortion by pregnant survivors of sexual violence is limited in such settings leading to many unintended pregnancies (6). In humanitarian situations, pro-delaying-pregnancy campaigns frequently ignore issues including limited access to contraception, lack of reproductive autonomy, and, in some cases, criminalized abortions. When transactional sex is utilized to relieve socioeconomic stress, the risk of sexually transmitted illnesses caused by sexual violence may increase (27, 30, 31).

## Conclusions

In humanitarian settings, there is the lack of quality data, and most studies are cross-sectional, which makes it extremely difficult to address causality. Most interventions implemented in these settings are inappropriate, as they are not grounded in any solid evidence (unethical). Obtaining parental consent/assent to carry out research among adolescents in humanitarian settings can be extremely difficult. It is another question knowing if true consent is easily obtainable in these settings, as persons might be intentionally or unintentionally “coerced” to participate in research (e.g., transport and time compensation, or fear of being named and not receiving services, especially if the research agenda is NGO driven). Researchers need to be particularly trained to understand the broader meanings of consent within these contexts. Ethics committees have their responsibilities to play. Indeed, the competence of research ethics committees in reviewing research protocols geared toward humanitarian settings needs to be strengthened. They can specifically seek to know how “true consent” will be sought from participants as a special requirement in the ethics applications. Conceptualization informed consent for humanitarian settings is an urgently needed but under explored area of research.

Research in humanitarian settings is essential and presents an opportunity to use setting-specific knowledge to improve practice. Hence, community engagement must be ensured through co-creating the research agenda from the inception, implementation, and evaluation of evidence-based interventions. Indeed, ownership, adaptation, and sustainability can only be achieved through effective community engagement in research in these settings. Core considerations like gender, ethics, and frameworks to adequately characterize context remain key. Ignoring effective community engagement predisposes to create new areas of inequalities, widen existing gaps, or aggravate the conditions of some already vulnerable groups. How communities in humanitarian settings should be effectively engaged in research is an important yet underexplored area of implementation science which is highly needed.

## Author contributions

LB, CN, PN, OB, LA, OA, ID, GM, and EK were contributed equally and significantly to its creation. LB read and made the necessary edits on the manuscript. All authors reviewed the final draft and gave their approval.

## References

[1] Bruno W Haar RJ. A systematic literature review of the ethics of conducting research in the humanitarian setting. Confl Health. (2020) 14:27. 10.1186/s13031-020-00282-0 32489418 PMC7245798

[2] Kohrt BA Mistry AS Anand N Beecroft B Nuwayhid I. Health research in humanitarian crises: an urgent global imperative. BMJ Global Health. (2019) 4:e001870. 10.1136/bmjgh-2019-001870 31798999 PMC6861060

[3] Singh NS Abrahim O Altare C. COVID-19 in humanitarian settings: documenting and sharing context-specific programmatic experiences. Confl Health. (2020) 14:79. 10.1186/s13031-020-00321-w 33292392 PMC7676860

[4] Leresche E Truppa C Martin C Marnicio A Rossi R Zmeter C. Conducting operational research in humanitarian settings: is there a shared path for humanitarians, national public health authorities and academics? Confl Health. (2020) 14:25. 10.1186/s13031-020-00280-2 32435274 PMC7222467

[5] Metuge A Omam LA Jarman E Njomo EO. Humanitarian led community-based surveillance: case study in Ekondo-titi, Cameroon. Confl Health. (2021) 15:17. 10.1186/s13031-021-00354-9 33771200 PMC7995751

[6] Civaner MM Vatansever K Pala K. Ethical problems in an era where disasters have become a part of daily life: a qualitative study of healthcare workers in Turkey. PLOS ONE 12:e0174162. 10.1371/journal.pone.0174162 28319151 PMC5358848

[7] Studying South Sudan's violence against women girls: ethical safety issues to take into account. Conflict and Health. Available online at: https://conflictandhealth.biomedcentral.com/articles/10.1186/s13031-019-0239-4 (accessed February 12, 2023).

[8] Meinhart M Vahedi L Carter SE Poulton C Palaku PM Stark L. Gender-based violence and infectious disease in humanitarian settings: lessons learned from Ebola, Zika, and COVID-19 to inform syndemic policy making. Confl Health. (2021) 15:84. 10.1186/s13031-021-00419-9 34801062 PMC8605893

[9] Leung MW Yen IH Minkler M. Community based participatory research: a promising approach for increasing epidemiology's relevance in the 21st century. Int J Epidemiol. (2004) 33:499–506. 10.1093/ije/dyh010 15155709

[10] Shanks L Moroni C Rivera IC Price D Clementine SB Pintaldi G. “Losing the tombola”: a case study describing the use of community consultation in designing the study protocol for a randomized controlled trial of a mental health intervention in two conflict-affected regions. BMC Med Ethics. (2015) 16:38. 10.1186/s12910-015-0032-x 26032480 PMC4450849

[11] Miller NP Ardestani FB Dini HS Shafique F Zunong N. Community health workers in humanitarian settings: scoping review. J Glob Health. (2020) 10:020602. 10.7189/jogh.10.020602 33312508 PMC7719274

[12] UNICEF-IRC. Humanitarian Research. UNICEF-IRC. Available online at: https://www.unicef-irc.org/research/humanitarian-research/ (accessed February 12, 2023).

[13] 5 Reasons Why We Need Research in the Humanitarian Sector. HAD. (2019). Available online at: https://had-int.org/blog/5-reasons-why-we-need-research-in-the-development-humanitarian-sector/ (accessed February 12, 2023).

[14] Bankoff G. Rendering the world unsafe: ‘vulnerability' as western discourse. Disasters. (2001) 25:19–35. 10.1111/1467-7717.00159 11244643

[15] Curchod C Patriotta G Cohen L Neysen N. Working for an algorithm: power asymmetries and agency in online work settings. Adm Sci Q. (2020) 65:644–76. 10.1177/0001839219867024

[16] Hülssiep M Thaler T Fuchs S. The impact of humanitarian assistance on post-disaster social vulnerabilities: some early reflections on the Nepal earthquake in 2015. Disasters. (2021) 45:577–603. 10.1111/disa.12437 32277843

[17] Thorneycroft R. Problematizing and reconceptualizing ‘vulnerability' in the context of disablist violence. In:Asquith NL Bartkowiak-Théron I Roberts KA, editor*s. Policing Encounters with Vulnerability* (Berlin: Springer), 27–46. 10.1007/978-3-319-51228-0_2

[18] Dada S Cocoman O Portela A Brún AD Bhattacharyya S Tunçalp Ö. What's in a name? Unpacking ‘Community Blank' terminology in reproductive, maternal, newborn and child health: a scoping review. BMJ Global Health. (2023) 8:e009423. 10.1136/bmjgh-2022-009423 36750272 PMC9906186

[19] Cyril S Smith BJ Possamai-Inesedy A Renzaho AMN. Exploring the role of community engagement in improving the health of disadvantaged populations: a systematic review. Glob Health Action. (2015) 8:29842. 10.3402/gha.v8.29842 26689460 PMC4685976

[20] Bain LE Akondeng C Njamnshi WY Mandi HE Amu H Njamnshi AK. Community engagement in research in sub-Saharan Africa: current practices, barriers, facilitators, ethical considerations and the role of gender - a systematic review. Pan Afr Med J. (2022) 43:152. 10.11604/pamj.2022.43.152.36861 36785694 PMC9922083

[21] Slim H Bonwick A. Protection: An ALNAP Guide for Humanitarian Agencies. Oxford: Oxfam (2006), p. 128. 10.3362/9780855988869

[22] Ford N Mills EJ Zachariah R Upshur R. Ethics of conducting research in conflict settings. Confl Health. (2009) 3:7. 10.1186/1752-1505-3-7 19591691 PMC2717053

[23] _ara.pdf. Available online at: https://apps.who.int/iris/bitstream/handle/10665/44783/9789290218814_ara.pdf (accessed February 12, 2023).

[24] Hunt MR. Establishing moral bearings: ethics and expatriate health care professionals in humanitarian work. Disasters. (2011) 35:606–22. 10.1111/j.1467-7717.2011.01232.x 21410748

[25] Gaglioti AH Werner JJ Rust G Fagnan LJ Neale AV. Practice-based Research Networks (PBRNs) bridging the gaps between communities, funders, and policymakers. J Am Board Fam Med. (2016) 29:630–5. 10.3122/jabfm.2016.05.160080 27613796 PMC5030066

[26] Yimer G Gebreyes W Havelaar A Yousuf J McKune S Mohammed A. Community engagement and building trust to resolve ethical challenges during humanitarian crises: experience from the CAGED study. Confl Health. (2020) 14:68 10.1186/s13031-020-00313-w 33042218 PMC7539377

[27] Akondeng C Njamnshi WY Mandi HE Agbor VN Bain LE Njamnshi AK. Community engagement in research in sub-Saharan Africa: approaches, barriers, facilitators, ethical considerations and the role of gender – a systematic review protocol. BMJ Open. (2022) 12:e057922. 10.1136/bmjopen-2021-057922 35545398 PMC9096545

[28] Contreras-Urbina M Blackwell A Murphy M Ellsberg M. Researching violence against women and girls in South Sudan: ethical and safety considerations and strategies. Confl Health. (2019) 13:55. 31832089 10.1186/s13031-019-0239-4 PMC6869273

[29] Zia Y Mugo N Ngure K Odoyo J Casmir E Ayiera E. Psychosocial experiences of adolescent girls and young women subsequent to an abortion in Sub-saharan Africa and globally: a systematic review. Front Reprod Health. (2021) 3:638013. 10.3389/frph.2021.638013 36303958 PMC9580653

[30] Mistry AS Kohrt BA Beecroft B Anand N Nuwayhid I. Introduction to collection: confronting the challenges of health research in humanitarian crises. Confl Health. (2021) 15:38. 10.1186/s13031-021-00371-8 33990200 PMC8120248

[31] Types of Disasters - Javatpoint. www.javatpoint.com. Available online at: https://www.javatpoint.com/types-of-disasters (accessed February 23, 2023).

